# Stimulating the Resolution of Inflammation Through Omega-3 Polyunsaturated Fatty Acids in COVID-19: Rationale for the *COVID-Omega-F* Trial

**DOI:** 10.3389/fphys.2020.624657

**Published:** 2021-01-11

**Authors:** Hildur Arnardottir, Sven-Christian Pawelzik, Ulf Öhlund Wistbacka, Gonzalo Artiach, Robin Hofmann, Ingalill Reinholdsson, Frieder Braunschweig, Per Tornvall, Dorota Religa, Magnus Bäck

**Affiliations:** ^1^Translational Cardiology, Karolinska Institutet, Stockholm, Sweden; ^2^Department of Cardiology, Theme Heart, Vessels, and Neuro, Karolinska University Hospital, Stockholm, Sweden; ^3^Theme Ageing, Karolinska University Hospital, Stockholm, Sweden; ^4^Department of Clinical Science and Education, Karolinska Institutet, Stockholm, Sweden; ^5^Department of Cardiology, Södersjukhuset, Stockholm, Sweden; ^6^Karolinska Trial Alliance, Sabbatsberg’s Hospital, Stockholm, Sweden; ^7^Department of Neurobiology, Care Sciences and Society, Karolinska Institutet, Stockholm, Sweden

**Keywords:** COVID-19, eicosanoids, omega-3 fatty acids, resolution of inflammation, clinical trial

## Abstract

Infection with severe acute respiratory syndrome coronavirus 2 (SARS-CoV-2) causes coronavirus disease 2019 (COVID-19). SARS-CoV-2 triggers an immune response with local inflammation in the lung, which may extend to a systemic hyperinflammatory reaction. Excessive inflammation has been reported in severe cases with respiratory failure and cardiovascular complications. In addition to the release of cytokines, referred to as cytokine release syndrome or “cytokine storm,” increased pro-inflammatory lipid mediators derived from the omega-6 polyunsaturated fatty acid (PUFA) arachidonic acid may cause an “eicosanoid storm,” which contributes to the uncontrolled systemic inflammation. Specialized pro-resolving mediators, which are derived from omega-3 PUFA, limit inflammatory reactions by an active process called resolution of inflammation. Here, the rationale for omega-3 PUFA supplementation in COVID-19 patients is presented along with a brief overview of the study protocol for the trial “Resolving Inflammatory Storm in COVID-19 Patients by Omega-3 Polyunsaturated Fatty Acids - A single-blind, randomized, placebo-controlled feasibility study” (COVID-Omega-F). EudraCT: 2020-002293-28; clinicaltrials.gov: NCT04647604.

## Introduction

Infection with severe acute respiratory syndrome coronavirus 2 (SARS-CoV-2) triggers an immune response and a local pulmonary inflammation which may extend to become a systemic inflammation. The leading cause of COVID-19 mortality is respiratory failure from acute respiratory distress syndrome (ARDS), which occurs in 15–29% of cases (Huang et al., 2020). Even in non-fatal cases the excessive inflammation can also extend to for example cardiovascular manifestations (Evans et al., 2020). While anti-inflammatory treatments are effective in dampening the acute inflammation, there may be a concern that this will cause an immunosuppression to aggravate infection. In this context, it is important to stress that the resolution of inflammation is an active process coordinated to limit and eventually turn off an inflammation while retaining an intact host defense (Panigrahy et al., 2020). Mediators of the resolution phase skew the immune response to clearance mechanisms of apoptotic cells, debris and microbes while limiting inflammatory activation (Serhan, 2014). This process actively promotes healing and repair and drives a tissue back to homeostasis after an acute infection and/or injury (Chiang and Serhan, 2020).

**FIGURE 1:**
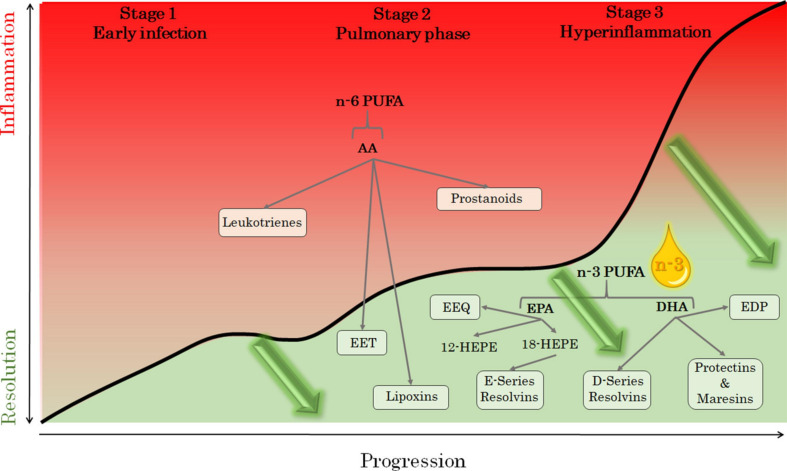
The metabolism of the omega-6 (n-6) and omega-3 (n-3) polyunstaurated fatty acids (PUFA) arachidonic acid (AA), eicosapentaenoic acid (EPA) and docosahexaenoic acid (DHA). The pathways depicted in red indicate proinflammatory mediators during the three stages of COVID-19 severity: early infection (stage 1), pulmonary phase (stage 2), and hyperinflammation phase (stage 3); adapted from Siddiqi and Mehra (2020). Intravenous administration of n-3 PUFA is anticipated to decrease n-6 PUFA and, in particular, AA-derived proinflammatory lipid mediators, and increase specialized pro-resolving mediators (including resolvins of the E- and D-series, protectins, and maresins), as well as the monohydroxy intermediates hydroxy-EPA (HEPE) and the cytochrome P450 epoxides epoxyeicosatrienoic (EET), epoxyeicosatetraenoic (EEQ), and epoxydocosapentaenoic (EDT) acids. Mediators potentially inducing the resolution of inflammation at the different stages of COVID-19 infection (green arrows), are depicted in green.

A three-stage classification has been proposed for COVID-19 to illustrate an initial mild infection, a second stage with established pulmonary involvement with or without hypoxia, and a third severe stage with hyperinflammation (Siddiqi and Mehra, 2020). Importantly, an active resolution of inflammation is needed to heal each stage of the disease, as depicted in Figure 1. A functional resolution of inflammation may also be crucial for presenting with only mild symptoms, whereas failed resolution may lead to escalating severity in clinical stages of COVID-19 (Figure 1). Importantly, the resolution of inflammation is impaired with ageing (Arnardottir et al., 2014), which may explain why younger people are less affected in COVID-19. In addition to age, the level of frailty is a predictor of short-term COVID-19 outcomes in older patient populations (Hägg et al., 2020).

Polyunsaturated fatty acids (PUFA) serve as the substrate for pro-inflammatory, anti-inflammatory, and specialized pro-resolving lipid mediators (SPM) (Chiang and Serhan, 2020). Specifically, the omega-6 PUFA arachidonic acid (AA) is substrate for the lipoxygenase and cyclooxygenase pathways, which generate leukotrienes and prostaglandins, respectively, collectively referred to as eicosanoids (Figure 1). In contrast, the omega-3 PUFA eicosapentaenoic acid (EPA) and docosahexaenoic acid (DHA) serve as the substrate for pro-resolving SPM (Figure 1). PUFA can also be metabolized by cytochrome (CYP) P450 epoxygenases into their respective epoxides (Figure 1), which also regulate the inflammatory reaction.

Increasing omega-3 PUFA and decreasing omega-6 PUFA hence represent a possible mean to skew the immune response toward resolution of inflammation (Figure 1). It should however, be considered that AA also gives rise to pro-resolving lipoxins, which is favored by the CYP450-derived AA epoxides (Hammock et al., 2020; Figure 1). In addition, another omega-6 PUFA, adrenic acid, has recently been ascribed anti-inflammatory actions (Brouwers et al., 2020). Nevertheless, a low omega-3 to omega-6 ratio is in general indicating a nutritional state favoring inflammation (Artiach et al., 2020b; Xue et al., 2020), which can also be reflected in the ratios of the respective lipid mediators, e.g., the resolvin to leukotriene ratio (Thul et al., 2017).

## Cytokine Storm in COVID-19

Increased levels of inflammatory cytokines triggered by SARS-CoV-2 in the peripheral blood cause an uncontrolled systemic inflammation, referred to as cytokine release syndrome or “cytokine storm” (Ragab et al., 2020). In particular, macrophage-related cytokines like interleukin (IL)-1β, IL-6, and tumor necrosis factor-α (TNF-α) were initially reported to be increased in COVID-19 infected patients compared with control subjects, with higher levels of cytokines in severe compared with non-severe infection (Chen et al., 2020; Huang et al., 2020; Ye et al., 2020). Furthermore, plasma concentrations of some cytokines were reported higher in COVID-19 patients admitted to intensive care units (ICU) compared with non-ICU patients (Chen et al., 2020; Huang et al., 2020). The degree of inflammation, measured as IL-6 plasma levels, correlates with the viral load determined as SARS-CoV-2 RNAemia (Huang et al., 2020). IL-6 is also a predictor of mortality for COVID-19 patients in ICU (Huang et al., 2020; Ruan et al., 2020). While both moderate and severe COVID-19 present with an increase in IL-6 (Chen et al., 2020), concentrations ≥100 pg/mL are observed exclusively in critically ill patients with high systemic viral nucleic acid detection (Huang et al., 2020). Taken together, these reports support that increased cytokine levels may be associated with worse clinical presentation and outcome in COVID-19, of which IL-6 may be an important driving force of the cytokine storm.

The concept of the cytokine storm has, however, been challenged recently. In a study of 46 patients with COVID-19-related ARDS, the levels of IL-6, IL-8, and TNF-α were lower compared with 51 SARS-CoV-2 negative patients with ARDS as a result of septic shock (Kox et al., 2020). Single cell sequencing has also failed to detect substantial amounts of pro-inflammatory cytokines in peripheral monocytes and lymphocytes from COVID-19 patients (Wilk et al., 2020). Nevertheless, pro-inflammatory, monocyte-derived macrophages are abundant in the bronchoalveolar lavage fluid of severe cases of SARS-CoV-2 infection (Liao et al., 2020), confirming the exaggerated inflammatory response in COVID-19. Taken together, the latter studies reinforce the importance of exploring non-cytokine mediators of inflammation as drivers of the COVID-19 inflammatory storm.

## Eicosanoid Storm in COVID-19

Eicosanoids derived from AA comprise the prostanoids [prostaglandins (PG) E_2_, PGD_2_, PGF_2α_, PGI_2_, and thromboxane (TXA_2_)], which are formed by the cyclooxygenase pathway, and the leukotrienes [LTB_4_ as well as the cysteinyl leukotrienes (cysLT) LTC_4_, LTD_4_, and LTE_4_], which are formed by the 5-lipoxygenase pathway (Figure 1). Eicosanoids contribute to inflammation by several mechanisms, e.g., recruitment of inflammatory cells (LTB_4_, PGD_2_), vasodilation (PGE_2_, PGI_2_), broncho- and vasoconstriction (PGE_2_, PGF_2α_, cysLT), hyperalgesia and pyrogenicity (PGE_2_), or increased vascular permeability (cysLT). Increased levels of pro-inflammatory lipid mediators have been described in uncontrolled immune responses to other severe infections and been coined “eicosanoid storm” (Dennis and Norris, 2015). A targeted liquid chromatography tandem mass spectrometry (LC-MS/MS) lipidomics analysis of serum from 18 moderate and 20 severe COVID-19 patients compared with 19 healthy subjects revealed major changes in AA-derived lipid mediators from both the cyclooxygenase and 5-lipoxygenase pathway, with severe COVID-19 being characterized by an increase in 5-lipoxygenase expressing monocyte/macrophage populations (Schwarz et al., 2020). These findings suggest that in addition to cytokines, an eicosanoid storm of pro-inflammatory lipid mediators may also contribute to the hyperinflammation in COVID-19 (Hammock et al., 2020).

Targeting pro-inflammatory eicosanoid signaling by means of the leukotriene receptor antagonist montelukast could have potential protective effects on pulmonary (Dahlén and Bäck, 2001), cardiovascular (Ingelsson et al., 2012), and inflammatory (Bäck et al., 2014) responses and is currently evaluated in COVID-19 (Pierantonio et al., 2020). Moreover, non-steroidal anti-inflammatory drugs (NSAID) suppress the formation of prostanoids and have been discussed to aggravate COVID-19 infection, but in the lack of evidence to support this notion, regular NSAID users are advised to continue with their therapy (FitzGerald, 2020). It can at present hence not be concluded if eicosanoid targeting during SARS-CoV-2 infection has either adverse or beneficial effects.

Importantly, supplementation with omega-3 PUFA decreases the relative availability of AA for pro-inflammatory eicosanoid synthesis and decreases leukotriene formation (Artiach et al., 2020a) and hence represents another possible means to decrease the eicosanoid storm in COVID-19.

## Resolution of Inflammation

As an alternative to inhibiting pro-inflammatory signaling, acute inflammation could potentially be disrupted by actively stimulating the resolution of inflammation. The enzymatic conversion of omega-3 PUFA into SPMs actively disrupts inflammatory circuits and skews the immune response toward healing and return to homeostasis (Serhan, 2014). Some SPM also inhibit viral replication and reduce the severity of viral pneumonia in experimental models (Morita et al., 2013; Duvall and Levy, 2016). Several observational and experimental studies also support a role of SPM in ARDS and acute lung injury (Darwesh et al., 2020).

The uncontrolled immune response observed in severe cases of COVID-19 with cytokine and eicosanoid release hence represents the archetype for a state of failure in the resolution of inflammation (Figure 1).

## Specialized Pro-Resolving Mediators in COVID-19

Lipidomic analysis has importantly showed that subjects with moderate COVID-19 exhibit significantly higher levels of the EPA-derived pro-resolving mediator RvE3 compared with severe cases (Schwarz et al., 2020). This observation provides a first indication that loss of pro-resolving mediators derived from omega-3 PUFA may be associated with more severe COVID-19. Furthermore, the resolvin biosynthetic pathways can be activated by SARS-CoV-2 viral proteins. Monocyte-derived macrophages isolated from subjects with cystic fibrosis and stimulated with the SARS-CoV-2 S and N proteins increase not only pro-inflammatory cytokines but also the DHA-derived pro-resolving lipid mediator RvD1 (Recchiuti et al., 2020). Increasing the substrates for E- and D series Rv by means of EPA and DHA supplementation hence has the potential to increase the formation of these pro-resolving mediators. Importantly, concomitant treatment of macrophages with SARS-CoV-2 viral proteins and exogenous RvD1 significantly reduces Macrophage Inflammatory Protein (MIP)-1α, TNF-α, and IL-8 (Recchiuti et al., 2020), further reinforcing the potential therapeutic benefit of increasing pro-resolving omega-3 PUFA-derived lipid mediators in COVID-19. Randomized controlled studies (RCT) have supported increased levels of pro-resolving mediators (Elajami et al., 2016) and a decreased inflammation (Mayer et al., 2003; Bhatt et al., 2019) after omega-3 PUFA supplementation.

## Anti-Inflammatory, Anti-Viral and/or Pro-Resolving Treatment Options in COVID-19

The sharp increase in numerous pro-inflammatory cytokines and its association to worse clinical presentation and outcome have led to the notion that targeting the cytokine storm may be an important part of rescuing severe COVID-19 patients (Mehta et al., 2020). Therapeutic options currently considered for this purpose include steroids, selective cytokine blockade, and other specific anti-inflammatory treatments (Mehta et al., 2020). Importantly, some anti-inflammatory therapeutic approaches are inherently characterized by a general immunosuppression, which may impede the host defense against the virus, and hence even aggravate the infection.

**FIGURE 2:**
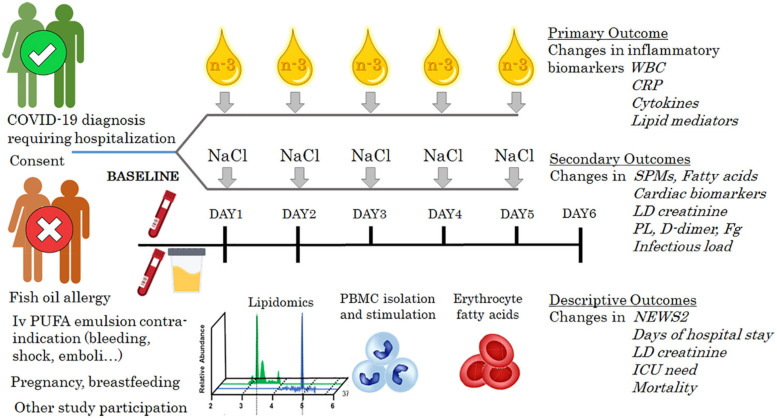
Schematic study protocol for the COVID-Omega-F trial, registered in the European Union Drug Regulating Authorities Clinical Trials (EudraCT) database with number 2020-002293-28 and at ClinicalTrials.gov (clinicaltrials.gov/ct2/show/NCT04647604). An intravenous omega-3 PUFA emulsion (n-3) is compared with placebo (NaCl) for effects on a panel of inflammatory biomarkers; white blood cells (WBC), C-reactive protein (CRP), cytokines and lipid mediators at the end of the treatment period. Secondary endpoints include changes in specialized proresolving mediators (SPMs), fatty acids, cardiac markers, lactate dehydrogenase (LD), D-dimer, fribronogen (Fg), and infectious load. National Early Warning Score 2 (NEWS2), length of hospital stay, intensive cae unit (ICU) need, and mortality will be monitored for a descriptive analysis. The inclusion criteria for COVID-Omega-F comprise female and male patients ≥18 years of age diagnosed with COVID-19 and presenting with a clinical status requiring hospitalization. Exclusion criteria are defined according to i.v. omega-3 PUFA emulsion contraindications, known hypersensitivity, participation in other clinical research study, pregnancy, and breastfeeding.

The omega-3 PUFA-derived pro-resolving mediator protectin D1 (PD1; Figure 2) attenuates influenza virus replication in experimental models (Morita et al., 2013). In influenza-infected mice, PD1 improves survival similar to antiviral treatment using peramivir starting 48 hr after infection. Interestingly, the combination of PD1 with peramivir completely rescued the mice from death due to the influenza infection (Morita et al., 2013). Although their clinical implications remain to be established, these preclinical observations indicate a potential additive effect between pro-resolving omega-3 PUFA-derived lipid mediators and antiviral treatment in preventing lethal infectious outcomes.

The benefit of glucocorticoid treatment in COVID-19 was demonstrated by the RECOVERY trial, showing lower 28-day mortality in ventilated and oxygen-treated patients randomized to either dexamethasone or usual care during hospitalization (Recovery Collaborative Group et al., 2020). Importantly, dexamethasone increases the production of pro-resolving lipid mediators from the omega-3 PUFA DHA (Figure 2), pointing to possible synergistic effects of omega-3 PUFA and cortisone treatment in COVID-19 (Pyrillou et al., 2018; Andreakos et al., 2020).

## Omega-3 PUFA Supplementation in ARDS

Significant improvements in ARDS patients have been reported after omega-3 PUFA administration. A recent meta-analysis of 12 RCT (*n* = 1280 patients) performed in patients with ARDS concluded that omega-3 PUFA supplementation was associated with improvements in the PaO_2_/FiO_2_ ratio, and statistical trends toward a shorter ICU stay (*p* = 0.08) and a shorter duration of mechanical ventilation (*p* = 0.06) were apparent, whereas infectious complications remained unchanged. In an analysis restricted to enteral omega-3 PUFA supplementation, a significant decrease in ARDS mortality was also seen (Langlois et al., 2019). A Cochrane meta-analysis of 10 studies noted a statistical reduction in mortality from ARDS when omega-3 PUFA were compared with a lipid-rich enteral formula but stated that it is currently uncertain whether omega-3 PUFA supplementation alters mortality, oxygenation, or duration of mechanical ventilation and ICU stays because of large heterogeneity between the studies (Dushianthan et al., 2019). There is hence a need for further studies of omega-3 PUFA supplementation in ARDS.

Omega-3 PUFA supplementation by means of intravenous (i.v.) administration provides an effective strategy of increasing omega-3 PUFA in the setting of acute disease and intensive care. One previous RCT has specifically evaluated the effects of i.v. omega-3 PUFA emulsion in ARDS (of other cause than COVID-19). Whereas the primary outcome of changes in respiratory parameters were not significantly altered in this RCT of 61 ventilated patients with ARDS, the observed fall in PaO_2_/FiO_2_ ratio from baseline to day 14 was significantly higher in the control group as compared to the *n* = 31 patients treated with i.v. omega-3 PUFA emulsion at 0.1 g/kg/day (Gupta et al., 2011). The latter study also reported a trend of better survival in the i.v. omega-3 PUFA emulsion group (77%) compared with the control group (56%; *p* = 0.10) (Gupta et al., 2011). Importantly, no adverse effects were observed, and no safety concerns of i.v. omega-3 PUFA emulsion treatment in ARDS were raised from this (Gupta et al., 2011) and other studies (Sabater et al., 2008), indicating that lipid emulsions enriched with omega-3 PUFA are safe in ARDS.

## Intravenous Omega-3 PUFA Supplementation and Inflammation

The rationale for studying i.v. omega-3 PUFA emulsion treatment in COVID-19 is strengthened by the possibility to reduce the inflammatory storm. In a study of 19 patients in septic shock, the 10 patients randomized to i.v. omega-3 PUFA emulsion (350 mL/day, which is equivalent to 14 g DHA + EPA) for 3 days attained an omega-3/omega-6 ratio of 2.5:1 and remarkably lower levels of TNF-α, IL-6, and IL-8 in *ex vivo* stimulated leukocytes (Mayer et al., 2003). Lower doses of omega-3 PUFA supplementation may, however, not be sufficient, as demonstrated by a larger study of different lipid emulsions in patients with systemic inflammatory response syndrome (SIRS), which resulted in an omega-3/omega-6 ratio of 1:7 in the control group and 1:2 in the supplementation group and did not show any significant differences in circulating IL-6 levels between the groups (Friesecke et al., 2008).

## Intravenous Omega-3 PUFA Supplementation in COVID-19

Based on the above, high dose omega-3 PUFA supplementation by the i.v. route appears as an appealing treatment option in COVID-19 with minimal risks to the patients. Taken together, these observations provide the rationale for actively stimulating the resolution of inflammation to break the inflammatory storm caused by SARS-CoV-2 infection. This is also in line with recent communications from other investigators on the potential benefits of enteral (Calder, 2020) and parenteral (Torrinhas et al., 2020) omega-3 PUFA supplementation in COVID-19 patients. Scientists in the field have stressed the uttermost priority to investigate pro-resolving lipid mediators in COVID-19 (Andreakos et al., 2020).

## The *COVID-Omega-F* Trial

To establish whether omega-3 PUFA supplementation by the i.v. route is a possible treatment option in COVID-19, we have initiated the trial “Resolving Inflammatory Storm in ***COVID***-19 Patients by ***Omega***-3 Polyunsaturated Fatty Acids – A single-blind, randomized, placebo-controlled ***F***easibility Study” (COVID-Omega-F). The study received approval from the National Ethics Board on May 25, 2020 (Dnr 2020-02592) and by the Medical Product Agency on May 29, 2020 (Dnr 5.1-2020-42861). Ten patients in each group will be randomized (in total *n* = 20). An amendment has been approved to increase inclusion up to *n* = 40 patients in total to achieve comparable groups completing the full study protocol according to the initial sample size calculations (National Ethics Board approval November 25, 2020; Dnr 2020-06137, and Medical Product Agency approval on November 30, 2020; Dnr 5.1-2020-96391). COVID-Omega-F is registered at the European Union Drug Regulating Authorities Clinical Trials (EudraCT) database with the number 2020-002293-28 and at ClinicalTrials.gov (clinicaltrials.gov/ct2/show/NCT04647604).

The study intervention is a single-blind randomization to daily administration of either an i.v. omega-3 PUFA emulsion containing 10 g of fishoil per 100 mL, of which 1.25–2.82 g DHA and 1.44–3.09 g EPA (0.2 g/kg/day at 0.5 mL/kg/h) or placebo (i.v. NaCl at 0.5 mL/kg/h to equivalent volume) for 5 days. The primary objective is to establish the effects of i.v. omega-3 PUFA emulsion on inflammatory biomarkers in COVID-19 patients compared to placebo. The primary endpoint is changes in a panel of inflammatory biomarkers measured in blood samples, urine samples, and released from *ex vivo* stimulated leukocytes at the end of the 5 day treatment period (Figure 2). The secondary endpoints are changes in pro-resolving mediators and PUFA levels, including the omega-3 to omega-6 ratio, in the erythrocyte fraction as well as measures of biomarkers for organ damage, thrombosis, and infectious load as indicated in Figure 2. Indicators for the clinical course of disease (Figure 2) will also be monitored during hospitalization and used for a descriptive analysis only since the study is not powered to detect clinical treatment benefits.

The inclusion criteria for COVID-Omega-F comprise female and male patients ≥ 18 years of age found COVID-19 positive or with typical CT image of COVID-19 infection, and clinical status requiring hospitalization. Exclusion criteria are defined according to i.v. omega-3 PUFA emulsion contraindications (serious bleeding disorders and acute life-threatening condition including acute shock, acute myocardial infarction, acute stroke, acute emboli, and coma), known hypersensitivity to the i.v. omega-3 PUFA emulsion or any of its ingredients, participation in any clinical research study evaluating an investigational medicinal product within 3 months prior to screening, pregnancy, and breastfeeding.

Blood samples will be collected for biomarker measures at inclusion before the first dose of treatment (baseline) and then daily until either completing 5 days of treatment or at hospital discharge (whichever comes first). At baseline, 24–48 h after the first dose of omega-3 PUFA emulsion or placebo infusion, and at treatment end, blood and urine samples will be collected for the following experimental procedures: Whole blood will be analyzed by flow cytometry for surface markers and functional assays. Peripheral blood mononuclear cells (PBMCs) will be isolated and stimulated with endotoxin followed by collection of supernatants for biomarker analysis and cells for real time quantitative PCR. The erythrocyte fraction will be used for determination of fatty acid composition by gas chromatography. Serum, plasma, urine, and PBMC supernatants will be used for a comprehensive analysis of inflammatory biomarkers, including cytokines and bioactive lipid mediators from the omega-3 and omega-6 metabolomes using LC-MS/MS. The lipid mediators to be detected include resolvins of the E and D series, lipoxins, leukotrienes, and prostanoids, as well as their intermediates (Figure 1), of which 18-HEPE has been previously established as a robust marker for pro-resolving mediator formation from omega-3 PUFA (Laguna-Fernandez et al., 2018).

## Conclusion

It is anticipated that i.v. omega-3 PUFA administration will decrease inflammatory mediators and that this will be indicative for potential beneficial clinical effects. Importantly, the simultaneous monitoring of pro-inflammatory and pro-resolving mediators will facilitate the understanding of a possible failure of the resolution of inflammation in COVID-19. In addition to obtaining the proof-of-concept for the resolution of inflammation through omega-3 PUFA treatment in COVID-19, this study will provide information on the feasibility of the study protocol. Together, this will lay the groundwork for a larger RCT on i.v. omega-3 PUFA administration on disease outcome in COVID-19.

## Author Contributions

All authors listed have made a substantial, direct and intellectual contribution to the work, and approved it for publication.

## Conflict of Interest

The authors declare that the research was conducted in the absence of any commercial or financial relationships that could be construed as a potential conflict of interest.
